# Supplementary material for: Histopathologic evaluation of postmortem autolytic changes in bluegill (Lepomis macrohirus) and crappie (Pomoxis anularis) at varied time intervals and storage temperatures
Source: PeerJ. 2016 Apr 19;4:e1943. doi: 10.7717/peerj.1943 (PMC4841231; doi:10.7717/peerj.1943)
Supplement: Data S1 — This file contains the original study design and the plan for processing the specimens used in the study. [file peerj-04-1943-s001.doc]

Tissue Collection Study

**Goal**

To harvest tissues from fresh, refrigerated and frozen fish held for various time periods so an assessment can be made on value of the samples presented for histology. We will be obtaining only normal tissues for this study.

**Day and Times of Harvesting**

**Tuesday, October 2^nd^**

8:00 am

- Euthanize, separately double bag 36 fish
  - 12 kept in container in room by the east tank (record room temperature)
  - 12 refrigerator
  - 12 put in freezer
- Euthanize 3 fish and immediately harvest needed tissues

12:00 pm (4 hours)

- Remove 3 fish from freezer
- Harvest tissues from 3 fish at room temperature
- Harvest tissues from 3 fish kept in refrigerator

1:00 pm

- Harvest tissues from 3 fish that had been frozen and then defrosted

8:00 pm (12 hours)

- Remove 3 fish from freezer
- Harvest tissues from 3 fish at room temperature
- Harvest tissues from 3 fish kept in refrigerator

9:00 pm

- Harvest tissues from 3 fish that had been frozen and then defrosted

**Wednesday, October 3^rd^**

8:00 am (24 hours)

- Remove 3 fish from freezer
- Harvest tissues from 3 fish at room temperature
- Harvest tissues from 3 fish kept in refrigerator

9:00 am

- Harvest tissues from 3 fish that had been frozen and then defrosted

**Thursday, October 4^th^**

8:00 am (48 hours)

- Remove 3 fish from freezer
- Harvest tissues from 3 fish at room temperature
- Harvest tissues from 3 fish kept in refrigerator

9:00 am

- Harvest tissues from 3 fish that had been frozen and then defrosted

**Wednesday, October 3rd**

8:00 am

- Remove 3 fish from freezer
- Harvest tissues from fish at room temperature
- Harvest tissues from fish kept in refrigerator

9:00 am

- Harvest tissues from fish that had been frozen and then defrosted

**Procedure for obtaining the tissues**

- Record the temperatures in the room working up the fish and the internal body temperatures for the fish.
- Open fish and remove all internal organs to side
- Obtain tissues in this order
  - Gill arch, upper and lower intestine, (the rest aren’t as critical as those two), but I typically do this: spleen, liver, stomach, gonad, gas bladder, posterior kidney, anterior kidney, heart, skin (include lateral line if possible), underlying muscle, brain, eye
