# Supplementary material for: Histopathologic evaluation of postmortem autolytic changes in bluegill (Lepomis macrohirus) and crappie (Pomoxis anularis) at varied time intervals and storage temperatures
Source: PeerJ. 2016 Apr 19;4:e1943. doi: 10.7717/peerj.1943 (PMC4841231; doi:10.7717/peerj.1943)
Supplement: Data S3 — This file contains details on tissue identification, contents of tissue cassettes, and scoring of the various organ samples. [file peerj-04-1943-s003.docx]

| **Histology ID #** | **Results** |
| --- | --- |
| F0a-1 |  |
| F0a-2 |  |
| F0a-3 |  |
| F0b-1 |  |
| F0b-2 |  |
| F0b-3 |  |
| F0c-1 |  |
| F0c-2 |  |
| F0c-3 |  |
| R4a-1 |  |
| R4a-2 |  |
| R4a-3 |  |
| R4b-1 |  |
| R4b-2 |  |
| R4b-3 |  |
| R4c-1 |  |
| R4c-2 |  |
| R4c-3 |  |
| R12a-1 |  |
| R12a-2 |  |
| R12a-3 |  |
| R12b-1 |  |
| R12b-2 |  |
| R12b-3 |  |
| R12c-1 |  |
| R12c-2 |  |
| R12c-3 |  |
| R24a-1 |  |
| R24a-2 |  |
| R24a-3 |  |
| R24b-1 |  |
| R24b-2 |  |
| R24b-3 |  |
| R24c-1 |  |
| R24c-2 |  |
| R24c-3 |  |
| R48a-1 |  |
| R48a-2 |  |
| R48a-3 |  |
| R48b-1 |  |
| R48b-2 |  |
| R48b-3 |  |
| R48c-1 |  |
| R48c-2 |  |
| R48c-3 |  |
| Z4a-1 |  |
| Z4a-2 |  |
| Z4a-3 |  |
| Z4b-1 |  |
| Z4b-2 |  |
| Z4b-3 |  |
| Z4c-1 |  |
| Z4c-2 |  |
| Z4c-3 |  |
| Z12a-1 |  |
| Z12a-2 |  |
| Z12a-3 |  |
| Z12b-1 |  |
| Z12b-2 |  |
| Z12b-3 |  |
| Z12c-1 |  |
| Z12c-2 |  |
| Z12c-3 |  |
| Z24a-1 |  |
| Z24a-2 |  |
| Z24a-3 |  |
| Z24b-1 |  |
| Z24b-2 |  |
| Z24b-3 |  |
| Z24c-1 |  |
| Z24c-2 |  |
| Z24c-3 |  |
| Z48a-1 |  |
| Z48a-2 |  |
| Z48a-3 |  |
| Z48b-1 |  |
| Z48b-2 |  |
| Z48b-3 |  |
| Z48c-1 |  |
| Z48c-2 |  |
| Z48c-3 |  |
| T4a-1 |  |
| T4a-2 |  |
| T4a-3 |  |
| T4b-1 |  |
| T4b-2 |  |
| T4b-3 |  |
| T4c-1 |  |
| T4c-2 |  |
| T4c-3 |  |
| T12a-1 |  |
| T12a-2 |  |
| T12a-3 |  |
| T12b-1 |  |
| T12b-2 |  |
| T12b-3 |  |
| T12c-1 |  |
| T12c-2 |  |
| T12c-3 |  |
| T24a-1 |  |
| T24a-2 |  |
| T24a-3 |  |
| T24b-1 |  |
| T24b-2 |  |
| T24b-3 |  |
| T24c-1 |  |
| T24c-2 |  |
| T24c-3 |  |
| T48a-1 |  |
| T48a-2 |  |
| T48a-3 |  |
| T48b-1 |  |
| T48b-2 |  |
| T48b-3 |  |
| T48c-1 |  |
| T48c-2 |  |
| T48c-3 |  |

**Microscopic Autolysis Scale:**

**Cassette 1**: gill arch and skin with skeletal muscle +/- scales

**Cassette 2**: kidney, liver, spleen, gonads, heart

**Cassette 3**: stomach, pyloric caeca, intestines, brain

- Null
  - No characteristics seen
  - 0%
- Rare
  - <1 /HPF
  - <1%
- Occasional
  - 1-3/HPF
  - 1-5%

Autolysis Characteristics:

- Cell edema/swelling
- Amorphous, dense deposits in the mitochondria
- Loss of glycogen granules
- Dilation of endoplasmic reticulum
- Clumping and margination of nuclear chromatin
- Condensation of nuclear chromatin
- Nuclear swelling
- Nuclear pleomorphism
- Intracytoplasmic vacuoles
- Abnormal characteristics for particular tissue sight Failure to take up stain (washed out)
- Pyknosis and karyolysis
- Cell membrane fragmentation
- Homogenous, eosinophilic cytoplasm
- Few
  - 4-10/HPF
  - 5-10%
- Moderate
  - 10-25/HPF
  - 10-50%
- Marked
  - >25/HPF
  - >50%

Tissue Sight and Normal Characteristics Of:

1. Gill arch
   1. Primary lamella and secondary lamellae
   2. Salt cells, RBCs, epithelial cells, endothelial cells, pillar cells
2. Skeletal muscle
3. Skin
   1. Includes the cuticle, epidermis, basement membrane, dermis (stratum spongiosum + stratum compactum), hypodermis, subcutaneous muscle (listed from superficial to deep)
   2. Epidermis is stratified squamous but capable of mitotic division arranged in a whirling pattern
      1. Contains malpighian cells (rounded), goblet cells (often in the center of the epidermis), club cells (secrete warning), granule cells, lymphocytes, MPs, large clear cyst-like structures
   3. Dermis – upper layer has loose network of collagen and reticulin fibers, pigment cells (chromatophores), mast cells, cells of the scale bed and scales; the lower layer has collagenous dense matrix for structure/strength of skin, melanophores, lipophores (erythrophores – red pigment and xanthopores – yellow pigment, leucophores and iridophores for white and silver)
   4. Hypodermis – looser adipose tissue that is highly vascular; * frequent site of infectious processes
4. Scale
   1. Ctenoid versus cycloid scales
   2. Growth rings on surface
   3. Collagen fibers interspersed with a matrix of albuminoid material (deposited hydroxyapatite crystals)
5. Kidney
6. Liver
7. Spleen
8. Heart
9. Gonads
10. Intestines
11. Pyloric Caeca
12. Stomach
13. Brain
